# Mitochondrial Fission-Mediated Lung Development in Newborn Rats With Hyperoxia-Induced Bronchopulmonary Dysplasia With Pulmonary Hypertension

**DOI:** 10.3389/fped.2020.619853

**Published:** 2021-01-28

**Authors:** Yuanyuan Dai, Binyuan Yu, Danyang Ai, Lin Yuan, Xinye Wang, Ran Huo, Xiaoqin Fu, Shangqin Chen, Chao Chen

**Affiliations:** ^1^Department of Neonatology, The Second Affiliated Hospital, Yuying Children's Hospital of Wenzhou Medical University, Zhejiang, China; ^2^Department of Neonatology, The Children's Hospital of Fudan University, Shanghai, China

**Keywords:** bronchopulmonary dysplasia, pulmonary hypertension, mitochondrial fission, Mdivi-1, Drp1, echocardiography, pulmonary vascular resistance

## Abstract

**Background:** Bronchopulmonary dysplasia (BPD) is the most common chronic respiratory disease in premature infants. Oxygen inhalation and mechanical ventilation are common treatments, which can cause hyperoxia-induced lung injury, but the underlying mechanism is not yet understood. Mitochondrial fission is essential for mitochondrial homeostasis. The objective of this study was to determine whether mitochondrial fission (dynamin-related protein 1, Drp1) is an important mediator of hyperoxia lung injury in rats.

**Methods:** The animal model of BPD was induced with high oxygen (80–85% O_2_). Pulmonary histological changes were observed by hematoxylin-eosin (HE) staining. Pulmonary microvessels were observed by immunofluorescence staining of von Willebrand Factor (vWF). Protein expression levels of Drp1 and p-Drp1 (Ser616) were observed using Western Blot. We used echocardiography to measure pulmonary artery acceleration time (PAT), pulmonary vascular resistance index (PVRi), peak flow velocity of the pulmonary artery (PFVP), pulmonary arteriovenous diameter, and pulmonary vein peak velocity. Mitochondrial division inhibitor-1 (Mdivi-1) was used as an inhibitor of Drp1, and administered through intraperitoneal injection (25 mg/kg).

**Results:** Pulmonary artery resistance of the hyperoxide-induced neonatal rat model of BPD increased after it entered normoxic convalescence. During the critical stage of alveolar development in neonatal rats exposed to high oxygen levels for an extended period, the expression and phosphorylation of Drp1 increased in lung tissues. When Drp1 expression was inhibited, small pulmonary vessel development improved and PH was relieved.

**Conclusion:** Our study shows that excessive mitochondrial fission is an important mediator of hyperoxia-induced pulmonary vascular injury, and inhibition of mitochondrial fission may be a useful treatment for hyperoxia-induced related pulmonary diseases.

## Introduction

Bronchopulmonary dysplasia (BPD) is a chronic lung disease that occurs in preterm infants who require respiratory support and oxygen therapy at birth (1). It is caused by a variety of molecular factors such as genetic predisposition, oxygen toxicity, and inflammatory injury, whose complex interactions are still not fully understood; and the prevention and treatment strategies for BPD are still limited (2–4). Impaired intrauterine lung development and post-partum injury can impair angiogenesis and alveolar formation, resulting in simplification of the distal alveoli. These characteristic histological changes of BPD clinically manifest as persistent respiratory diseases, requiring long-term oxygen supplementation (5), and pulmonary hypertension (PH). Approximately 15–25% of BPD cases will develop PH (6). Among severe cases of BPD, the incidence of PH is higher (7). Furthermore, the existence of PH is closely related to adverse outcomes of BPD, and the mortality rate of BPD combined with PH is as high as 48% (8).

It is generally believed that mitochondrial dynamics play a vital role in mitochondrial homeostasis (9). Mitochondrial fission is mediated mainly by dynamin-related protein 1 (Drp1), a GTPase associated with cytoplasmic dynamin-related proteins, which belongs to the dynamin-related family and was the first fission protein discovered (10). When activated, cytoplasmic Drp1 is transported to the mitochondrial outer membrane, where GTPase is hydrolyzed and polymerized (9). Accumulating data also suggest that Drp1 is a key molecule in mitochondrial dynamics that controls mitochondrial fusion and fission (11), and abnormal expression of it may lead to abnormal changes in chronic lung diseases such as PH and lung cancer (12, 13). Besides, post-translational modification of Drp1, such as phosphorylation at Ser616, is an important mechanism for modulating mitochondrial fission (14). Recent studies have found that hypoxia can lead to mitochondrial fission of pulmonary artery smooth muscle (15), but the changes in pulmonary vascular mitochondrial dynamics induced by excessive oxygen have not been studied.

Echocardiography is a common method of PH examination in adults and children (16). Echocardiography can show direct signs of PH due to increased tricuspid regurgitation. However, the tricuspid regurgitation velocity used in adult pulmonary artery pressure estimations cannot be used in children, particularly infants, because it is difficult to obtain a good and clear image, and this measurement may not have good agreement with the data measured using a cardiac catheter (17). Therefore, indirect signs, such as changes in right ventricular function and changes in pulmonary artery acceleration time, are indispensable.

In this study, we hypothesized that hyperoxia would induce mitochondrial fission and thus impact lung development, resulting in the occurrence of BPD combined with PH. We found that after excessive oxygen stimulation, alveolar simplification, PH, and p-Drp1 mitochondrial translocation increased mitochondrial fission. Mdivi-1 is a Drp1 inhibitor that decreases mitochondrial fragmentation (18). Our results also suggest that inhibition of mitochondrial fission may be a useful treatment strategy for hyperoxia-associated pulmonary endothelial injury and related diseases.

## Materials and Methods

### Hyperoxia-Induced Lung Injury

All animal experiments were performed in accordance with the policies and guidelines of the Laboratory Animal Ethics Committee of Wenzhou Medical University. A total of 10 pregnant Sprague Dawley rats were purchased from the Experimental Animal Center of Wenzhou Medical University. The dams were maintained in humidity- and temperature-controlled rooms on a 12:12-h light-dark cycle and were allowed food and water *ad libitum*. On the final day of pregnancy, the dams delivered naturally (120 pups). Seventy-two pups from six pregnant rats were pooled, randomized, and returned to the nursing dams and then divided into two groups: the control (*n* = 36) and hyperoxia (*n* = 36) groups. The hyperoxia group of pups was exposed to 80–85% oxygen in a sealed Plexiglass box for 14 days, while the control group was maintained in room air (21% oxygen). Over the 14 days, the nursing dams were exchanged between the two groups every 24 h to avoid oxygen toxicity. The oxygen level of the Plexiglass box was monitored continuously using an oxygen analyzer.

The pups from the other four pregnant rats (48 pups) were experimentally divided into four groups: control + vehicle (*n* = 12), control + Mdivi-1 (*n* = 12), hyperoxia + vehicle (*n* = 12), and hyperoxia + Mdivi-1 (*n* = 12). Mdivi-1 (25 mg/kg) was given to the pups on days 7–14 by intraperitoneal injection. The pups in the control + vehicle and hyperoxia + vehicle groups were injected with the same volume of vehicle (corn oil, Sohrab Biotechnology, Beijing, China).

### Lung Histology and Morphometric Analyses

After 14 days, all hyperoxia groups (hyperoxia alone, hyperoxia + vehicle, and hyperoxia + Mdivi-1) were maintained in room air. Sixty pups in total from the control and hyperoxia groups were sacrificed on days 3, 7, 14, 21, and 28 by injection of 1% pentobarbital. The left lungs were removed and fixed in 4% paraformaldehyde for 48 h. The sections were then embedded into paraffin and sliced into 4-μm sections for hematoxylin-eosin (HE) staining (Sohrab Biotechnology, Beijing, China). At the same time, the right lungs were stored at −80°C for western blot.

The radial alveolar count (RAC, the number of alveoli contained in the terminal respiratory unit), which reflects the degree of alveolation, and Mean alveolar diameter (MAD) was the average alveolar diameter (19). And they were important indicators for the evaluation of non-development. Briefly, six lung sections were taken for HE staining on days 3, 7, 14, 21, and 28 from the control and hyperoxia groups, five fields were randomly selected for imaging under a 100x magnification lens, and the number of alveoli passing from the center of the respiratory bronchioles to the nearest interpleural line were counted as the RAC. The MAD was measured by Image-Pro Plus 6.0 software (Media Cybernetics, Rockville, MD, USA).

### Immunofluorescence

On day 14, the lung tissue sections from 24 rats [control + vehicle (*n* = 6), control + Mdivi-1(*n* = 6), hyperoxia + vehicle (*n* = 6), and hyperoxia + Mdivi-1 (*n* = 6)] were dried overnight at 37°C and then hydrated in xylene and an ethanol gradient series. The sections were then heated in a microwave in 10 mM citric acid buffer (pH 6.0) for 20 min for antigen retrieval. The sections were then incubated in 5% bovine serum albumin at 37°C for 1 h. The sections were incubated at 4°C overnight with a rabbit polyclonal anti-vWF (AF3000; 1:200 dilution; Affinity Biosciences. OH. USA), while the negative control group was incubated with phosphate-buffered saline. The sections were then incubated with Alexa Fluor-594 sheep anti-rabbit IgG (AB150076; diluted 1:500; Abcam) at room temperature for 4 h and subsequently with 4′,6-diamidino-2-phenylindole (DAPI). Dual immunophotography images were acquired using a scanning microscope (C1; Nikon, Tokyo, Japan).

### Western Blotting

Protein was extracted from frozen lung tissue samples from 36 rats in the control and hyperoxia groups on days 3, 7, and 14, and mixed with the loading buffer. Equal amounts of protein were separated by 10% sodium dodecyl sulfate-polyacrylamide gel electrophoresis at 100 V for 3 h and transferred to polyacrylamide difluoride membranes at 100 V for 50 min. Membranes were then blocked in 5% skimmed milk for 3 h at room temperature (20–25°C). Membranes were then incubated with rabbit monoclonal anti-Drp1 (ab184247; 1:500 dilution; Abcam, Cambridge, UK) or rabbit Phospho-Drp1 (Ser616) Antibody (#3455; 1:1,000 dilution; Cell Signaling Technology, Boston, USA) and gently shaken at 4°C overnight. The next day, the membranes were incubated with horseradish peroxidase-conjugated goat anti-rabbit or anti-mouse secondary antibody (1:5,000 dilution; Cell Signaling Technology, Boston, USA) for 2 h after being washed three times in Tris-buffered saline and tween-20, and developed using enhanced chemiluminescence reagents (Thermo Scientific Pierce; Thermo Fisher Scientific, Waltham, MA, USA). Densitometry values of each sample were calculated by Image Lab 5.0 software (Bio-Rad, Hercules, CA, USA) for all bands and standardized relative to β-actin.

### Echocardiographic Imaging

On days 14, 21, 28, and 42, six pups from the control and hyperoxia groups and 24 rats from other four groups on days 21 and 28 were prepared for ultrasonic imaging. The rats were anesthetized with isoflurane using a small animal respiratory anesthesia machine (R620-S1, RWD life science, Shenzhen, China). After full chest hair removal, the rats received continuous isoflurane anesthesia, were fixed in the supine position on the examination table, and connected with electrocardiogram electrodes. Their chests were coated with coupling agent.

The probe was slightly adjusted upwards to obtain a minor axial view of the aorta, and the probe was slightly tilted cephalically to obtain a major axial view of the pulmonary artery. The left atrial pulmonary vein junction was located, and the Doppler sampling point was placed at the junction to measure the pulmonary vein flow velocity.

To measure the hemodynamics of the pulmonary arteries and veins, the short-axis view of the aortic valve was obtained first, and the pulmonary artery was identified using a color Doppler instrument. The diameter of the pulmonary artery was measured at the attachment point of the pulmonary valve. The pulsed Doppler gate was placed at the proximal end of the pulmonary valve at an incident angle <20° to maximize laminar flow. Pulmonary acceleration time (PAT), pulmonary ejection time (PET), and peak flow velocity of the pulmonary artery (PFVP) were measured. PAT was measured as the time from the beginning of systolic blood flow to the peak flow rate, while PET was measured as the time from the beginning of systolic blood flow to the completion of pulmonary blood flow. The pulmonary vascular resistance index (PVRi) was calculated as the ratio of PET to PAT. Similarly, the size of the left atrial pulmonary vein junction was measured as the value of the pulmonary vein diameter, and the peak velocity of the pulmonary vein was measured according to the pulmonary vein flow velocity curve. To measure right ventricular load, short-axis views of the right and left ventricles were obtained at the level of the distal mitral valve.

Right and left ventricular diastolic area (RVEDA and LVEDA, respectively) were measured by manual endocardial boundary tracing. The ratio between RVEDA and LVEDA (RVEDA/LVEDA) was calculated as the measurement index of the right ventricular load (20).

### Statistical Analysis

The experiments were performed in triplicate and repeated at least three times. The data are presented as the mean ± SD or SEM and were analyzed using one-way analysis of variance (ANOVA) followed by Tukey's *post-hoc* test (equal variance) or Dunnett T3's *post-hoc* test (unequal variance) for multiple comparisons. Correlation analyses were performed using Spearman's rank correlation. Statistical analysis was carried out using SPSS Statistics 19.0 (SPSS Inc., Chicago, USA) or GraphPad 6.0 (GraphPad Software, San Diego, USA). Values of *P* < 0.05 were considered statistically significant.

## Results

### Hyperoxia Stunts Alveolar Development, Which Is Restored After Recovery in Room Air

**Figure 1:**
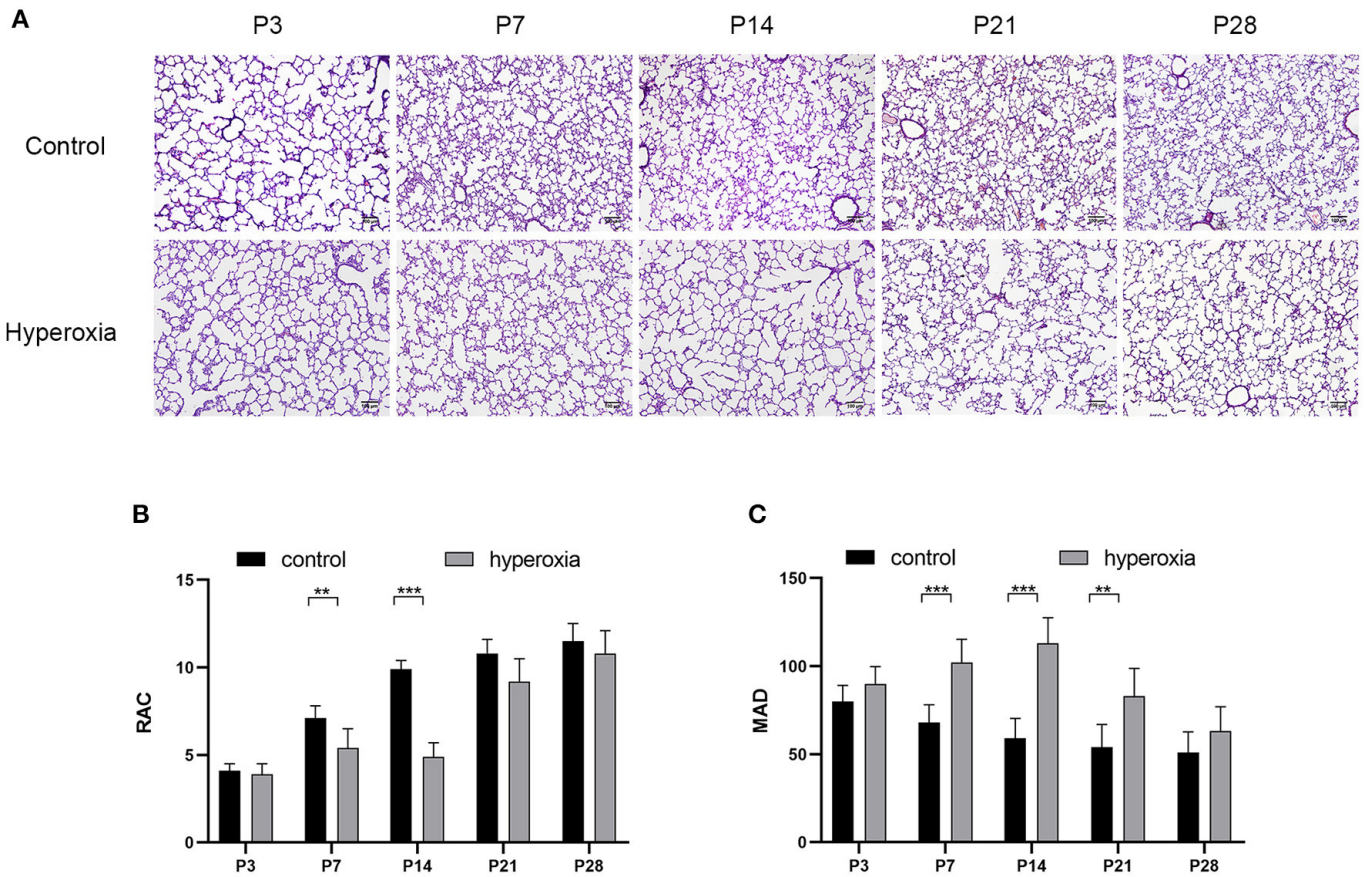
Morphological changes in rat lungs after hyperoxia. Newborn pups (P0) were exposed to 21% O_2_ (control) or 80–85% O_2_ (hyperoxia) for 14 days. **(A)** Hematoxylin and eosin staining of rat lungs exposed to hyperoxia or control group on day 3, 7, 14, 21, and 28. Scale bar: 100 μm. **(B)** Compared with the controls, the radial alveolar counts (RACs) of hyperoxia group were significantly reduced on five periods. **(C)** The Mean alveolar diameter (MAD) of hyperoxia group were significantly increased on five periods. *n* = 6 per group. Data are shown as mean ± SD; **P < 0.01, ***P < 0.001.

Hyperoxia causes substantial morphological simplification in the lung tissue, including a visible decrease in alveolar numbers and increase in alveolar size. These negative changes in the hyperoxia group can be quantified by the decrease in RAC and MAD, an important indicator of lung development. Significant histological differences between the hyperoxia and control groups were found in day 3, 7, 14, 21, and 28 (Figure 1A). This result indicated that chronic exposure to hyperoxia interrupts alveolar formation, as evidenced by a decrease in alveolar numbers and increase in alveolar diameter. On day 7 (Figure 1B), the RAC number of control group was higher than hyperoxia group (*P* < 0.01), and on day 14 this number in control group was much higher than in hyperoxia group (*P* < 0.001). However, these developmental abnormalities in the hyperoxia group were reversed after recovery in room air for 14 days. As for the MAD (Figure 1C), the MAD value of control group was significantly lower than hyperoxia group on day 7 (*P* < 0.001), on day 14 (*P* < 0.001), and on day 21 (*P* < 0.01).

### Hyperoxia Causes Drp1 Overexpression in Newborn Rat Lungs

**Figure 2:**
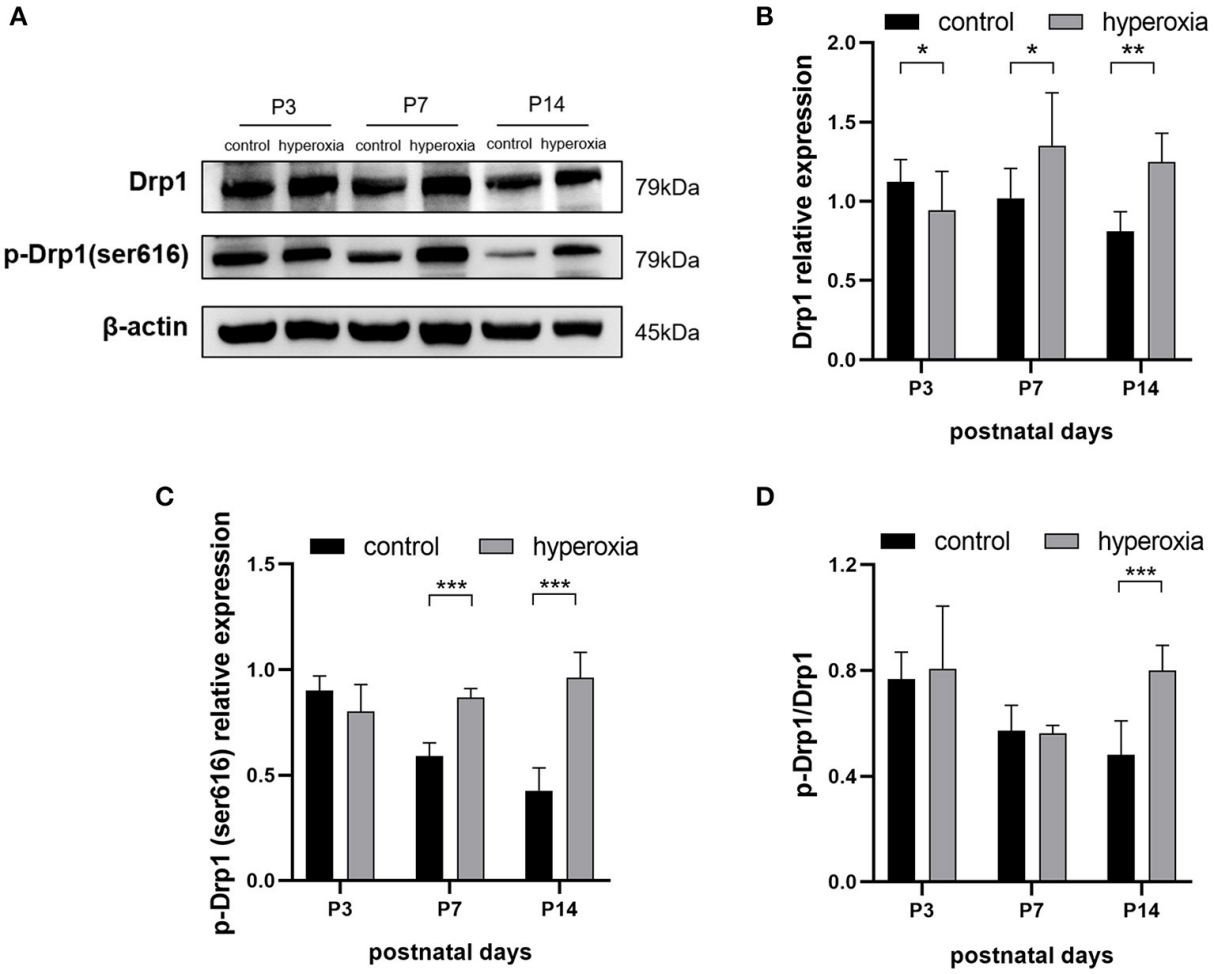
The levels of DRP1 and p-DRP1 (Ser616) in total lung tissues are detected by Western blotting. **(A)** Representative Western blot images of DRP1 and p-DRP1 (Ser616) in lung tissues from controls or from hyperoxia group on day 3, 7, or 14. **(B,C)** Protein levels of DRP1 **(B)** or p-DRP1 (Ser616) **(C)** in arbitrary units (AU) normalized to β-actin levels. **(D)** The ratios of p-DRP1 (Ser616)/DRP1 was calculated based on Western blot results. β-actin was the loading control. *n* = 6 animals/group; Values are expressed as means ± SD; *P < 0.05, **P < 0.01, ***P < 0.001.

To investigate whether hyperoxia altered the expression of Drp1 in rat lung tissues, we examined the levels of Drp1 and p-Drp1 over time by western blotting (Figure 2A). On the third day of hyperoxia (Figure 2B), the expression of Drp1 in the hyperoxia group was higher than that in the control group (*P* < 0.05). On the seventh day of hyperoxia, the levels of Drp1 (*P* < 0.05) and p-Drp1 (Ser616) (*P* < 0.001) in the hyperoxia group were both significantly elevated (Figures 2B,C), although the ratios of p-Drp1 (Ser616)/Drp1 were not different between the two groups, suggesting that the elevated p-Drp1 (Ser616) levels increased concomitantly with Drp1 levels (Figure 2D). After 14 days of hyperoxia, Drp1 levels in the hyperoxia group were still high (*P* < 0.01). Furthermore, the ratio of p-Drp1 (Ser616) to Drp1 (*P* < 0.001) was significantly increased compared to the controls, indicating that the phosphorylation of Drp1 at Ser616 (Figure 2C) was actively upregulated (*P* < 0.001).

### Pharmacological Inhibition of Drp1 With Mdivi-1 During Hyperoxia Mitigates Pulmonary Vascular Complications

**Figure 3:**
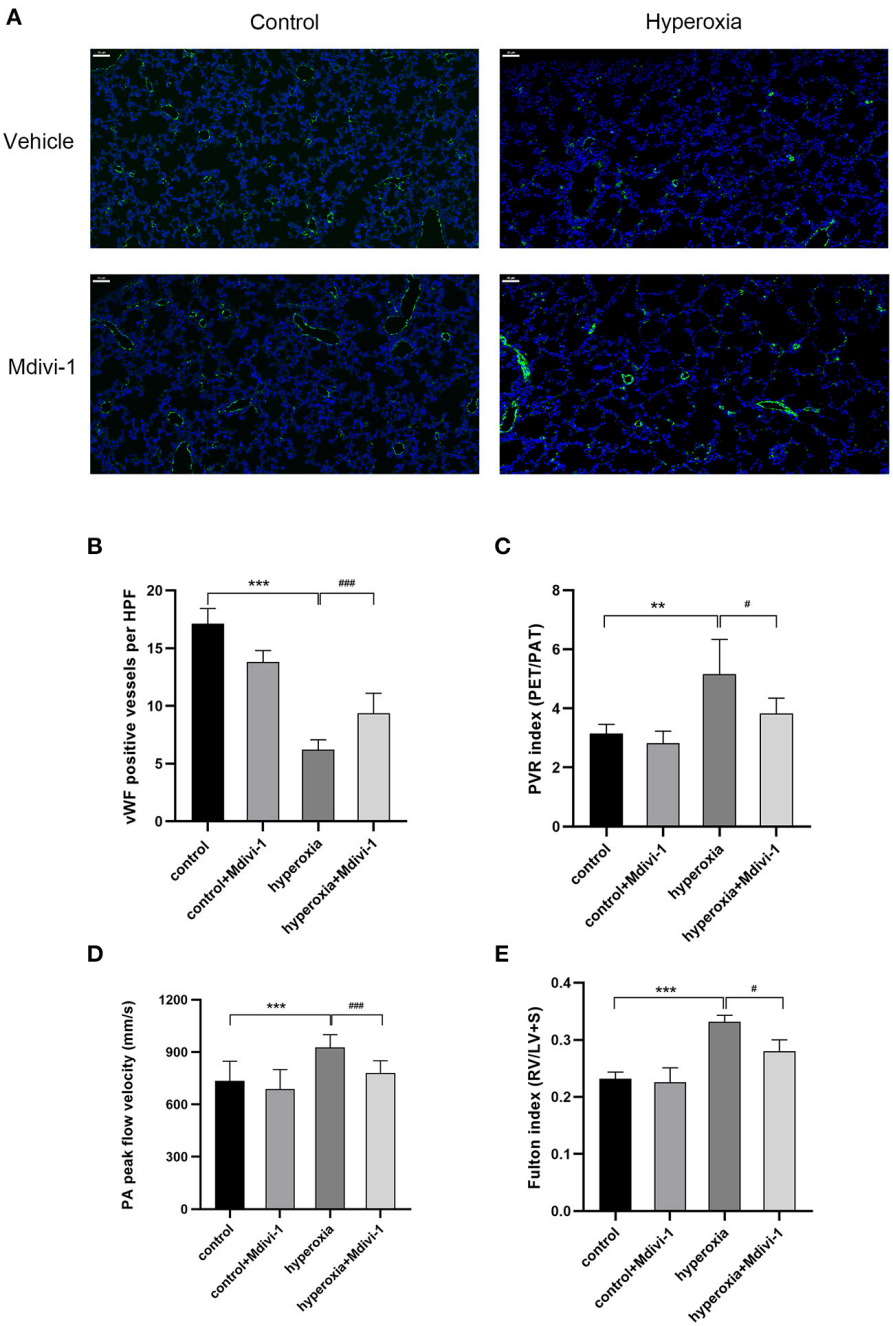
The beneficial effect of Mdivi-1 on pulmonary vasculature after chronic hyperoxia-induced lung injury. DRP1 inhibitor Mdivi-1 was injected intraperitoneally daily to the rats from day 7 to 14. **(A)** Representative images of immunofluorescence staining. Green fluorescence represented vWF expression. Scale bar = 50 μm. **(B)** vWF-positive vessels whose diameters were < 50 μm were calculated accordingly. **(C)** The pulmonary vascular resistance index (PVRi) measured on day 21. **(D)** The peak pulmonary flow velocity was measured on P28 **(E)** the Fulton's index was measured on day 28. *n* = 6 animals/group; Values are presented as mean ± SD. **P < 0.01, ***P < 0.001, hyperoxia vs. control; ^#^*P* < 0.05, ^*###*^*P* < 0.001, hyperoxia+Mdivi-1 vs. hyperoxia.

Mdivi-1 is a specific inhibitor of Drp1. Based on the results that DRP1 started to increase in the hyperoxia group on P7 and the high level persisted until P14, the Drp1 inhibitor, mdivi-1, was injected intraperitoneally daily from days 7 to 14 to investigate whether mdivi-1 had protective effects on chronic hyperoxia-induced lung injury. To evaluate vascular development, vWF-positive small blood vessels were visualized using immunofluorescence staining and counted (Figure 3). We found that long-term hyperoxia significantly decreased the number of pulmonary small blood vessels at day 14 (*P* < 0.001), whereas mdivi-1 treatment in the hyperoxia group (Figure 3B) partially rescued this decrease at day 14 (*P* < 0.001), indicating that mdivi-1 can alleviate hyperoxia-induced obstruction of pulmonary vascular development. To evaluate the long-term effects on blood vessels, ultrasonic echocardiogram monitoring revealed that the pulmonary vascular resistance index (Figure 3C) of the hyperoxia + mdivi-1 group was significantly lower than that of the hyperoxia + vehicle group on P21 (*P* < 0.05). The peak pulmonary flow velocity was measured on P28 (Figure 3D). The results showed that the pulmonary artery peak flow velocity decreased after administration of mdivi-1, indicating an improvement in pulmonary artery pressure. In addition, the heart tissue was weighed and Fulton index was calculated on P28. The Fulton index of the hyperoxia + vehicle group was significantly higher than that of the control + vehicle group (Figure 3E), indicating that the right ventricle was hypertrophic. Compared to the hyperoxia + vehicle group, the Fulton index of the hyperoxia + mdivi-1 group was lower, suggesting that right ventricular hypertrophy had improved (Figure 3E).

### Recovery in Room Air Leaves Hyperoxia-Exposed Rats With Abnormal Pulmonary Hemodynamics Until Adolescence

**Figure 4:**
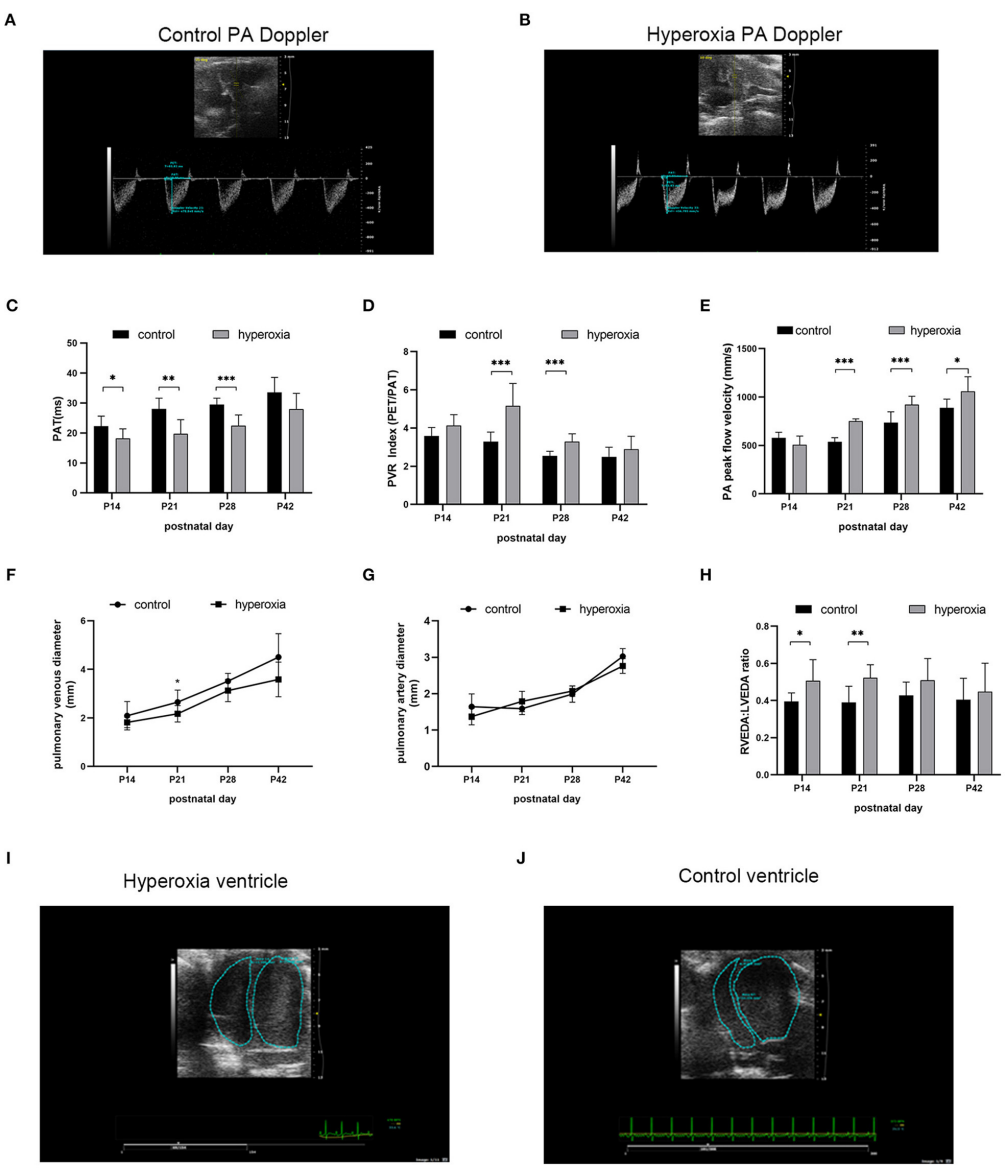
Abnormal pulmonary hemodynamics after hyperoxia. Newborn (P0) pups were exposed to 21% O_2_ (control) or 80–85% O_2_ (hyperoxia) for 14 days and returned to air to receive echocardiography detection on post-natal day 14, 21, 28, and 42. **(A)** Representative Doppler ultrasound images of the pulmonary artery (PA) on day 21 after exposure to either normoxia or hyperoxia for 14 days from birth. **(B)** Representative 2-dimensional echocardiography images of left and right ventricular dimensions on day 21 was shown by comparing the difference between exposure to normoxia and hyperoxia for 14 days after birth. **(C–H)** Echocardiography and pulse-wave Doppler-derived indexes of pulmonary acceleration time (PAT) **(C)**, pulmonary vascular resistance (PVR) index **(D)**, peak flow velocity of pulmonary artery **(E)**, pulmonary venous diameter **(F)**, pulmonary artery diameter **(G)**, and right ventricular end-diastolic area (RVEDA)-to-left ventricular end-diastolic area ratio (LVEDA) **(H)** were analyzed and compared between hyperoxia group and control group on P14, P21, P28, and P42. **(I,J)** Representative two-dimensional echocardiography images of the left and right ventricular dimensions on control and hyperoxia group on day 21. *n* = 6 animals/group; Values are expressed as means ± SD; *P < 0.05, **P < 0.01, ***P < 0.001, compared with hyperoxia and control group.

Although the alveolar developmental obstruction caused by hyperoxia had no significant difference in lung morphology between the control and hyperoxia groups after 7 days of normal oxygen recovery, the pulmonary vessels showed significant abnormalities in the hyperoxia group during the recovery period. We performed continuous pulmonary vascular-related cardiac ultrasonography on rats released from the hypertoxic environment on post-natal day 14, P21, P28, and P42 (Figure 4). After measuring indexes from the Doppler ultrasound trace of the pulmonary artery (Figures 4A,B), the results showed that the PAT (Figure 4C) reflecting the pulmonary circulation resistance was markedly shortened on day 14 (*P* < 0.05), day 21 (*P* < 0.01), and day 28 (*P* < 0.001). The other two indicators: PVRi and peak pulmonary flow velocity were significantly increased (Figures 4D,E). PVRi were significantly increased on day 21 (*P* < 0.001) and day 28 (*P* < 0.001) in the hyperoxia group compared to the control group. and the peak pulmonary flow velocity were increased on day 21 (*P* < 0.001), day 28 (*P* < 0.001), and day 42 (*P* < 0.05). In addition, cardiac ultrasound-related features of the pulmonary veins were also detected. After hyperoxia, the diameter of the pulmonary vein (Figure 4F) decreased on day 21 (*P* < 0.05), whereas the diameter of the pulmonary artery (Figure 4G) was not significantly changed at day 21. In order to observe the effect of this pulmonary hemodynamic abnormality on the heart, particularly on the right ventricular load, we acquired a short-axis image of the end-diastolic ventricle at the mitral valve level, and outlined the cross-sections of the right and left ventricular cavities. The area ratio (RVEDA/LVEDA) is a measurement of the right ventricular load. We found that the area ratio was higher in the hyperoxia group on day 14 (*P* < 0.05) and on day 21 (*P* < 0.01), suggesting that the right ventricle was dilated, and this trend continued for 7 days (up to day 21) in the hyperoxia group (Figure 4H). A representative two-dimensional echocardiography image of the left and right ventricular dimensions on day 21 demonstrated right ventricular dilation in the hyperoxia group compared to the control group, indicative of diastolic right ventricle dysfunction (Figures 4I,J).

## Discussion

In this study, we attempted to explore the relationship between BPD and mitochondrial fission induced by hyperoxia. To simulate severe BPD in rodent models, we first exposed rats to 80–85% oxygen for a long period (14 days). The results showed that the lung morphology was seriously damaged and alveolar structure was simplified, which was consistent with the pathology of BPD. Protein expression of Drp1/p-Drp1 in the hyperoxia group was significantly higher compared to in the control group. We then applied the Drp1 inhibitor, Mdivi-1, and found an improvement in the reduction of pulmonary microvasculature under hyperoxia. Finally, we discussed whether hyperoxia-induced BPD would have adverse effects on pulmonary circulation function and followed up with echocardiography.

In recent years, many studies have shown that mitochondrial dysfunction plays an important role in BPD and PH (21). Mitochondrial dynamics are essential for maintaining mitochondrial integrity and regulating apoptosis (22). Drp1 is a mitochondrial outer membrane protein that mediates fission of mitochondria and controls mitochondrial morphology (23). The latest research shows that by inducing overexpression of hypoxia inducible factor-1, hypoxia stimuli can promote the expression of Drp1 to regulate mitochondrial dynamics in pulmonary vascular remodeling (24). In this study Drp1 changes were investigated after hyperoxia exposure in newborn rats to further our understanding of the relationship between hyperoxia and Drp1. We found that Drp1 reached its peak on day 7 in the hyperoxia group and maintained this level until day 14. These results showed that Drp1 protein expression could be enhanced by hyperoxia. Many studies have suggested that abnormally high expression of Drp1 in the lungs is an indicator of poor prognosis, particularly in chronic malignant diseases (25, 26). Drp1 has been regarded as an attractive therapeutic target.

Mitochondrial oxidative stress is a component of general oxidative stress, and excessive reactive oxygen species would lead to increased mitochondrial fission (27). Studies have found that particulate matter (PM_2.5_) can lead to oxidative stress in lung epithelial cells, increasing mitochondrial fission, resulting in cell apoptosis (28), and Drp1 and oxidative stress are essential mediators in cigarette smoke-induced pulmonary endothelial Injury (29). Combined with our findings, we speculated that lung injury caused by hyperoxia in newborn rats would increase mitochondrial fission, namely the expression of Drp1, due to oxidative stress. In addition, experiments have confirmed the relationship between hypoxia and Drp1, studies were not only involved in animal models about Lung Ischemia-reperfusion Injury (30) and lung vascular ischemic/hypoxic injury (31), but also in others like hepatocellular carcinoma cells in hypoxia (32), and Hypoxia-Reoxygenation Injury of Cardiomyocytes (33). Although hypoxia or hyperoxia can trigger similar pathological responses, such as oxidative stress and inflammation, these underlying mechanisms need to be further studied at the cellular level.

Mdivi-1, a specific inhibitor of DRP1 (34) is reported to be effective in suppressing the pulmonary artery smooth muscle cells in lungs with PH (35). Because of the changes in Drp1 after hyper oxygen in this study, the rats were injected intraperitoneally with Mdivi-1 (25 mg/kg) from days 7 to 14 to explore whether inhibition of Drp1 has protective effects on hyperoxia-induced lung injury. It was found that long-term hyperoxia severely hindered the development of small pulmonary vessels, and after mdivi-1 administration, the number of small pulmonary vessels significantly increased. These results indicate that Mdivi-1 can relieve hyperoxia-induced obstruction of pulmonary microvascular development. From the perspective of the long-term effects on blood vessels, ultrasonic monitoring results showed that the PVR and PFVP measured during the recovery period were lower after the administration of Mdivi-1, suggesting improvements in pulmonary artery pressure. In addition, the heart tissue was weighed on 28 days after birth to calculate the Fulton index the results showed that the right ventricular hypertrophy index in the hyperoxia group was significantly higher than that in the control group, suggesting right ventricular hypertrophy.

To investigate whether this BPD model would have an adverse effect on pulmonary circulation function, follow-up detection was carried out by echocardiography in rats at 14, 21, 28, and 42 days after birth. It was demonstrated that PAT in the hyperoxia group was shortened, while the PVRi and PFVP increased significantly compared to the control group. These parameters all reflected higher pulmonary pressure after exposure to hyperoxia for 2 weeks. In addition, by measuring the area ratio and Fulton index, we found that over-circulation influenced right ventricular structure and function.

It is worth mentioning that in this study, in addition to focusing on the pulmonary artery, cardiac echocardiography indicators related to the pulmonary vein were also detected, and it was found that the pulmonary vein diameter showed signs of narrowing after exposure to hyperoxia, while there was no significant difference in pulmonary artery diameter between the two groups. Pulmonary vein stenosis is a rare problem that is often neglected (36); however, it is a severe and increasingly common complication of preterm infants with BPD (37). Although this study identified the manifestations of pulmonary vein stenosis, it did not elucidate the underlying mechanisms, which require further investigation.

Lastly, there are some limitations present in this study. First, we did not further explore the mechanism between hyperoxia and Drp1. Secondly, we did not carry out in *vitro* experiments, such as on pulmonary epithelial cells and microvascular endothelial cells. Thirdly, this experiment only discussed the development of pulmonary vessels after hyperoxia stimulation, but future studies will focus on the effects of Drp1 and Mdivi-1 on alveolar development.

In conclusion, the present study identified the echocardiographic features of hyperoxia-induced BPD-PH models and confirmed that the expression of Drp1 is increased in hyperoxia-induced lung injury. Treatment with Mdivi-1 during hyperoxia was protective against pulmonary vasculature development and function. However, further studies are required to determine the precise mechanism of Drp1 in BPD and BPD-PH.

## Data Availability Statement

The raw data supporting the conclusions of this article will be made available by the authors, without undue reservation.

## Ethics Statement

The animal study was reviewed and approved by Wenzhou Medicial University.

## Author Contributions

YD conceived and designed the experiments. YD and BY performed the experiments and wrote the paper. YD, DA, and LY analyzed the data. XW, RH, and XF contributed materials and analysis tools. CC and SC edited and approved final draft. All authors contributed to the article and approved the submitted version.

## Conflict of Interest

The authors declare that the research was conducted in the absence of any commercial or financial relationships that could be construed as a potential conflict of interest.
